# Effect of the Host on Deep-Blue Organic Light-Emitting Diodes Based on a TADF Emitter for Roll-Off Suppressing

**DOI:** 10.3390/nano9091307

**Published:** 2019-09-12

**Authors:** Manish Kumar, Luiz Pereira

**Affiliations:** 1 Department of Physics and i3N—Institute for Nanostructures, Nanomodulation and Nanofabrication, University of Aveiro, 3810-193 Aveiro, Portugal; mkumar@ua.pt; 2 CeNTI—Centre for Nanotechnologies and Smart Materials, R. Fernando Mesquita, 2785, 4760-034 Vila Nova de Famalicão, Portugal

**Keywords:** OLEDs, TADF, host, DMOC-DPS, roll-off

## Abstract

To achieve significant efficiency and low roll-off in thermally activated delayed fluorescence (TADF) material organic light-emitting diodes (OLEDs), it is essential to choose a host that has suitable high triplet energy (T_1_) and bipolar character to boost the TADF characteristics as a dopant and avoid exciton annihilation. Herein, we present the effect of different host materials on the efficiency of organic light-emitting diodes (OLEDs) based on bis[4-(3,6 dimethoxycarbazole)phenyl]sulfone (DMOC-DPS) deep-blue emitter. The devices with 10 wt.% of an emitter in different electron types of host bis[2-(diphenylphosphino) phenyl] ether oxide (DPEPO), and hole types of host 1,3-bis(*N*-carbazolyl)benzene (mCP), were fabricated to study the effect on device performance. The results show that an external quantum efficiency (EQE) of 4% and maximum current efficiency (ƞ_c_) up to 5.77 cd/A with high luminescence (l_max_) 8185 cd/m^2^ in DPEPO was achieved, compared to 2.63% EQE, ƞ_c_ 4.12 cd/A with l_max_ 5338 cd/m^2^ in mCP in a very simple device structure. As a remarkable result, the roll-off is suppressed at 1000 cd/m^2^, and for maximum brightness, the roll-off is less than 50%. Further general applications are discussed.

## 1. Introduction

The organic light-emitting diodes (OLEDs) field has been booming in past decades, and it continues to grow in both research and technology. More than ever, the use of organic materials in LEDs is recognized as a critical breakthrough, and the science of studying these organic materials is thriving. The main principle behind OLED technology is electroluminescence, and such devices offer brighter, thinner, high contrast, and flexible emitters [1].

The thermally activated delayed fluorescence (TADF) process has recently emerged as one of the most attractive methods for harvesting both singlet and triplet states in metal-free organic materials for application in highly efficient OLEDs because they can achieve a theoretical maximum internal quantum efficiency (IQE) up to 100% [2,3,4,5,6,7,8]. In TADF materials, the triplet excitons (T_1_) are readily upconverted to the singlet state (S_1_) by virtue of the reverse intersystem crossing (RISC) due to their near-zero singlet-triplet splitting (ΔE_ST_) [3,6,8,9,10]. Therefore, all S_1_ excitons emit light via a prompt or delayed fluorescence [7,11]. Despite the significant progress made in recent years, considerable challenges persist to achieve a full understanding of the TADF mechanism, and, importantly, to improve the stability of the devices based on such emitters, and to suppress the well-known roll-off.

To date, many deep-blue TADF emitters have been reported for their use in various applications with a maximum external quantum efficiency (EQE) over 30% [2,3,7,9,12,13]. Generally, deep-blue TADF emitters possess high triplet energy T_1_ over 2.8 eV [3]. However, in blue TADF emitters, a long-delayed exciton lifetime and strong intermolecular charge transfer (ICT) [6,11] leads to a broad emission spectrum due to the exciton-polaron quenching and triplet-triplet annihilation (TTA) with consequent high roll-off [7,11,14,15,16,17]. Therefore, for such emitters, our main objective is, in active layer, to confine all singlet and triplet excitons and avoid the TTA effect. This implies that specific hosts properties are required. First, the hosts should have T_1_ around or above 3.0 eV to prevent exciton quenching [6]. Second, it is also essential that in a host-guest system, the host should ensure the excitons transfer to the guest [2,3,6,8,17]. Third, suitable charge carrier transporting abilities are essential to increase the chances of the hole and electron recombination within the emitting layer [18]. Therefore, in TADF based OLEDs, the host material plays a more vital role in increasing the efficiency and suppressing the roll-off, compared to phosphorescent OLEDs [19,20].

At present, many studies of blue TADF emitters have been carried out in different n-type or p-type hosts, such as host bis[2-(diphenylphosphino) phenyl] ether oxide (DPEPO), 2,8-bis(diphenylphosphine oxide) dibenzofuran (DBFPO), 4,4′-bis(*N*-carbazolyl)-1,1′-biphenyl (CBP), and 1,3-bis(*N*-carbazolyl)benzene (mCP) [3,7,8,21,22,23]. In a carbazole based host such as CBP (mCP with the optimum T_1)_, the carbazole unit acts as a hole transfer unit, while in a phosphine–oxide based host (i.e., DPEPO), the phosphine unit acts as an electron transport unit [2,8]. In phosphine–oxide based hosts, the narrow-induced emission zone arises owing to unbalanced charge transportation, which increases the probability of TTA [24]; while in carbazole unipolar hosts, the lower triplet-triplet resonance between both host and guest due to the lower T_1_ (<3.0 eV) impacts overall device performance. Therefore, using such hosts for a suitable device is not simple. Nevertheless, some good results have reported that the final device structure is very complex, sometimes with more than five organic layers, which is a technological constraint for practical applications [2,3]. Decreasing the blue OLED structure complexity should be one of the fundamental objectives.

As for OLED practical applicability, the high roll-off is a severe constraint [25,26]. This issue can be eliminated by using multiple electron and hole transport layers, but this will increase the fabrication complexity and low reproducibility, and such problems constantly persist for the blue TADF emitters. Considering these questions, it is worthwhile to accept that a balance between efficiency/brightness must be achieved, finding the best compromise (regardless the absolute values) to keep efficiency as constant as possible in all the dynamic voltage range of OLED operations (i.e., suppressing the roll-off as much as possible). From the literature, the published data report a very high EQE, but this is only achieved at applied voltages immediately after the turn-on voltage; as the applied voltage increases slightly, the EQE decreases quickly, sometimes more than two orders of magnitude, which is a bottleneck for applications.

To attempt to address this issue, we demonstrated the effect of the host environment versus device structure on overall performance with suppressed roll-off. The device was fabricated in the two usual hosts (mCP and DPEPO) with a very simplified structure. We have studied blue DMOC-DPS TADF dopant in a hybrid solution thermally processed device with 10 wt.% doping of TADF to understand the efficient charge transfer from host to TADF dopant with further competitive exciton emission/annihilation for final enhanced device performance. For the first time, we show the best roll-off for a blue TADF emitter found in the literature is a very simple structure. An explanation about different device characteristics in both the hosts is given, along with a comparison with earlier-reported results of the same TADF.

## 2. Materials and Methods

### 2.1. Device Fabrication

**Figure 1:**
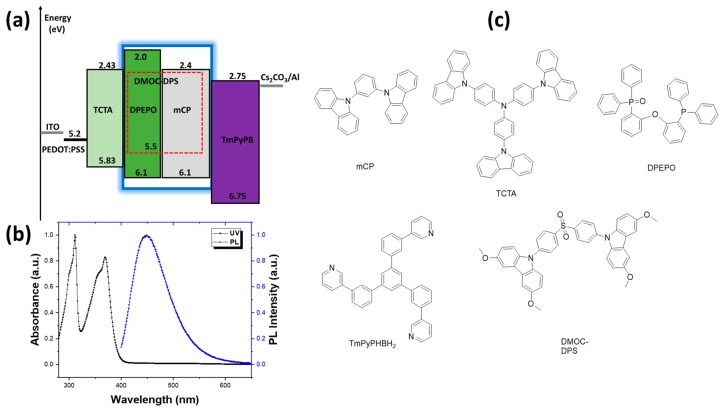
(**a**) Energy schematic of the device, and (**b**) UV-Vis an photoluminescence (PL) spectra of bis[4-(3,6 dimethoxycarbazole)phenyl]sulfone (DMOC-DPS) in toluene (the concentration was 1 mM, λ_ex_ at 350 nm at room temperature), and (**c**) chemical structures of the materials used in this work.

We fabricated multilayer OLEDs using deep-blue DMOC-DPS TADF dopant in different hosts DPEPO and mCP as the emitting layer (EML). This TADF was first reported by Adachi et al. in 2013 [27]. The device structure is ITO/PEDOT:PSS (40 nm) (Poly(3,4-ethylenedioxythiophene)-poly(styrenesulfonate))/TCTA (10 nm) (Tris(4-carbazoyl-9-ylphenyl)amine)/EML (20 nm)/TmPyPb (40 nm) (1,3,5-Tri(m-pyridin-3-ylphenyl)benzene)/Cs_2_CO_3_ (2 nm)/Al (100 nm) (Figure 1a), (see molecular structures in Figure 1c). The substrates were cleaned in an ultrasonic bath containing 1% *v*/*v* Hellmanex solution in water, acetone, and 2-propanol (IPA). Before the fabrication, substrates were treated with UV Ozone treatment for 5 min. The PEDOT:PSS was first filtered with a 0.45 μm PVDF (Polyvinylidene Fluoride) filter and then spin-coated. Both PEDOT:PSS and TCTA were deposited via a solution-processed method, while EML, TmPyPb, Cs_2_CO_3_, and Al were thermally deposited. A 40 nm PEDOT:PSS layer was spin-coated at 2000 rpm and annealed at 120 °C for 15 min. Then, 10 nm TCTA layer (chlorobenzene solvent) was spin-coated at 1500 rpm after being filtered using a 0.1 μm PTFE filter and dried in the glove box at 70 °C for 30 min. Here, TCTA was used as a hole injection layer, and TmPyPB was used as an electron injection layer. The emissive layer was then thermally deposited. DMOC-DPS was co-evaporated with the hosts to ensure the optimum 10 wt.% concentration in the EML. The rate of the deposition was 1 Å/s for organic materials, 0.1 Å/s for Cs_2_CO_3_, and 3 Å/s for Al. The active device area was 4.5 mm^2^. The doping concentration of TADF dopant DMOC-DPS was optimized to 10 wt.%. The energy diagram of TADF and hosts are shown in Figure 1a. All the materials were purchased from Luminescence Technology Corp. (Lumtec), Taipei, Taiwan.

### 2.2. Optical and Electrical Characterization

The current density–voltage–luminance (J–V–L) characteristics were determined using a Keithley Source-Meter 2425 model (Tektronix Inc., Beaverton, OR, USA). For the EL measurements, an Ocean Optics USB4000 spectrometer (Ocean Optics Inc.,Florida, FL, USA) was used with the sensitivity response in the wavelength range 350–950 nm. Luminance (L) was recorded using a Minolta LS-100 chromameter (Konica Minolta Inc., Tokyo, Japan). Photoluminescence (PL) of studied samples in toluene were performed at room temperature with an Edinburgh Instruments FLS980 fluorescence spectrometer (Edinburgh Instruments Ltd., Livingstone, UK) with Xe-lamp as an excitation source and R-928 photomultiplier detector.

## 3. Results and Discussion

### 3.1. UV-Vis Absorbance and Photoluminescence (PL) Characteristics

Figure 1b shows the absorption and PL spectra of DMOC-DPS in toluene at room temperature. In absorption, two bands at 310 nm and 370 nm were observed, which corresponds to a typical DMOC-DPS moiety absorption. In the emission spectra of DMOC-DPS, a wideband at 450 nm was observed, which is assigned to an intramolecular charge transfer transition from the methoxy-substituted carbazoles into the diphenyl sulfone [27]. DMOC-DPS exhibits the first singlet (S_1_; 3.1 eV), and triplet (T_1_; 2.88 eV) excited states with the lower energy gap between S_1_ and T_1_ (ΔE_ST_; 0.38 eV) [27]. For indication, the HOMO levels of DPEPO and mCP are similar (6.1 eV), whereas the LUMO level of DPEPO is 0.4 eV higher (2.0 eV) than that of mCP (2.4 eV) due to the electron-donating phosphine moieties.

### 3.2. Device Characteristics

**Figure 2:**
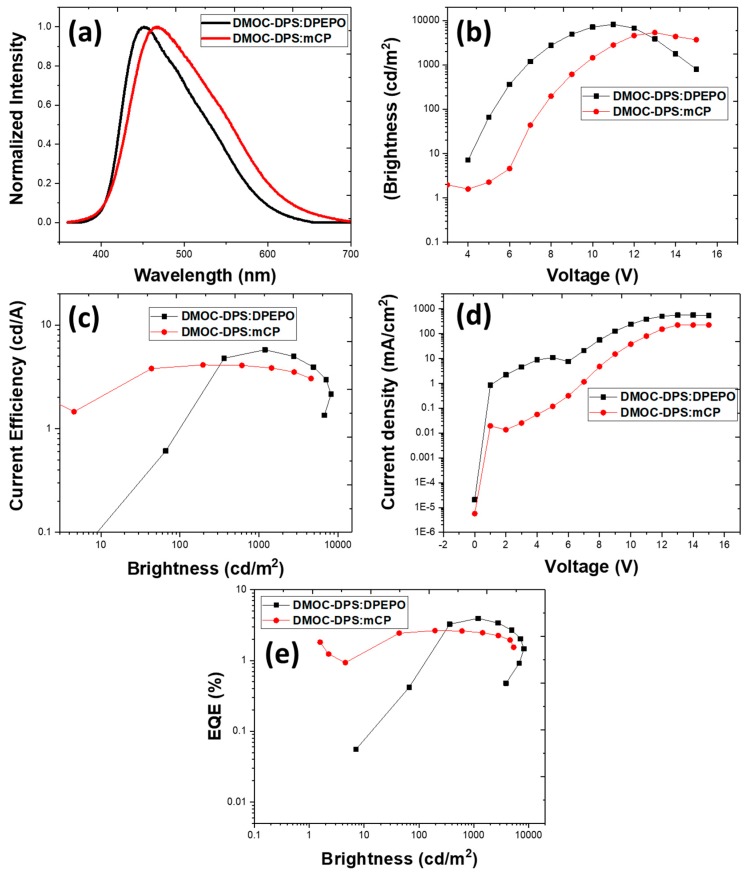
The device characteristics of the thermally activated delayed fluorescence (TADF) material based organic light-emitting diodes (OLED): (**a**) Electroluminescence spectra of both the devices at 8 V, (**b**) brightness-voltage curves, (**c**) current efficiency-brightness curves, (**d**) current density-voltage curve, and (**e**) EQE-brightness curves.

The J–V–L and EL characteristics of OLEDs from mCP and DPEPO hosts are shown in Figure 2. The EL spectrum (Figure 2a) of both the devices was almost identical to the PL of the DMOC-DPS in toluene. The devices with DPEPO host exhibited a peak at 450 nm with a blue color where the Commission Internationale de L’Eclairage (CIE) coordinates are (0.18, 0.23) (Figure 2b), whereas the device with the mCP host exhibited EL peak at 470 nm. The results with DPEPO are like the PL spectra in toluene, but there was a red shift in mCP host by 20 nm, with (CIE) coordinates of greenish-blue (0.21, 0.29). No additional emission was observed in the EL spectrum of both the devices, which indicates that only the TADF emission occurs without any other radiative recombination.

Usually, the host polarity is responsible for a shift to the TADF emission. This process is generally known as solvatochromism effect and was initially observed in photophysics studies of emitters in solution [28,29,30]. Although originally related to emitters in solution, solvatochromism can also be observed in solid-state films. It is currently called solid-state solvation (SSS) [31,32] in that it depends on the matrix polarizability [33]. The dielectric constant (ε) of the DPEPO and mCP is 6.12 and 2.84, respectively [34]. However, the EL spectra of all the results lead to a conclusion that the position of PL/EL maxima of the guest-host systems decorrelates with the host polarity. This is in agreement with the previous report [34]. The host-polarity effect on the charge transfer (CT) energies state varies very little, thus constituting a minor effect for the RISC process and the final TADF emitter efficiency [17,35].

Besides the polarity question, perhaps the more important question is whether the use of a host-guest matrix in the emissive layer will modulate the device behavior. The HOMO/LUMO energy levels and electrical carrier mobility in the emissive layer can face a noticeable change. From the different charge carrier mobilities, we can expect a shift in the balance of electron/hole densities in the active layer. The location of energy levels will influence the charge injection/transport and finally the performance. The mCP host has a high hole and electron mobility (4 × 10^−4^ cm^2^ V^−1^ s^−1^ and 2 × 10^−4^ cm^2^ V^−1^ s^−1^, respectively) [36], while the electron mobility of DPEPO is low (7.03 × 10^−8^ cm^2^ V^−1^ s^−1^ [37]) and expecting the same for the hole (is an n-type material). The current density characteristics are shown in Figure 2b–d.

The devices based on DMOC-DPS achieved a maximum current efficiency 5.77 cd/A in the DPEPO host compared to 4.12 cd/A in the mCP host, attributed firstly to a better efficient exciton harvesting, which results from T_1_→S_1_ upconversion. The EQE values were 4% and 2.63% for DPEPO and mCP devices, respectively. The device with DPEPO turned on (V_ON_) at 4 V with l_max_ of 8185 cd/m^2^, compared to V_ON_ of 3 V, l_max_ of 5338 cd/m^2^ with mCP. The lower V_ON_ in mCP can be attributed to the less carrier blocking effect at TCTA/emissive layer and emissive layer/TmPyPB interfaces as compared to the DPEPO host-based devices, although in both kinds of OLEDs, these values are of the lowest found for a simplified deep-blue emitter device. Both the devices showed (comparatively in the literature) good EQE at 1000 cd/m^2^ (i.e., 4% for DPEPO as hosts and 2.5% for mCP as hosts), but the most important result is an excellent roll-off suppressing characteristic that remains constant at higher brightness values. Even at maximum brightness, both the devices showed excellent EQE of 2% and 1.53% for DPEPO and mCP as hosts, respectively, which was the one of best behaviors found for this specific blue TADF emitter. The power efficiency (ƞ_P_) was 2.6 lm/W and 1.70 lm/W in DPEPO and mCP hosts, respectively. For now, it is clear that the host matrix of DPEPO enhances the TADF emission for high brightness, which is significantly attractive in this device structure. Moreover, the device with DPEPO as host exhibited excellent results. All the main results are summarized in Table 1.

It is worth pointing out that this specific TADF is particularly hard to use as an OLED emitter with simultaneously suitable efficiency/roll-off. For instance, Ban et al. [38] also employed DMOC-DPS as an emitter. Although the authors reported a better maximum current efficiency, compared with our results, the high roll-off persists. The maximum ƞ_c_ ~ 11 cd/A was obtained for L < 1 cd/m^2^ but at L ~ 1000 cd/m^2^, ƞ_c_ is only close to 0.2 cd/A showing the question of high roll-off. In our devices, the maximum ƞ_c_ ~ 5.8 cd/A was obtained for L ~ 1000 cd/m^2^, and at L ~ 2000 cd/m^2^ it was constant. Even at maximum l_max_ > 8500 cd/m^2^, we still have a ƞ_c_ of ~4 cd/A. We must stress that our devices have only three organic layers (PEDOT:PSS acting as anode optimization) with a lover V_ON_ < 4V. The other experimental result by Adachi et al. [27] for the same DMOC-DPS emitter exhibits high efficiencies but not good brightness (L_max_ of 2544 cd/m^2^). It is worthwhile to consider that the high EQE was obtained for L < 0.1 cd/m^2^, but for L = 100 cd/m^2^ is near 1.5%. Nevertheless, the device was a complex six organic layered structure, and the roll-off was very high with a high V_ON_.

All these results clearly exhibit the main problem regarding efficiency/roll-off. Moreover, no suitable solution has been reported that simultaneously overcame the problem and kept the device structure simple enough for more attractive practical applications. As referred before, TTA is the typical main problem for noticeable roll-off. As the carrier injection density increases, as expected, an increase in brightness is observed due to the higher rate of radiative recombination, but the quenching at excited levels become more pronounced leading to a marked decrease of the EQE. Particularly problematic is the case of emitting materials exhibiting delayed fluorescence. In TADF, as the long lifetime of excited electrical carriers increases, the probability of TTA increases dramatically [39]. Theoretically, it is possible to overcome this issue, if, for high current density range, the exciton density in the active layer is high enough for high brightness but moderate as required for avoiding TTA or quenching. The emitter concentration easily controls this late; TTA should be, from an electrical point of view, controlled by the carrier density profile in the active layer. The exciton profile in the active layer naturally depends on the electron/hole profile arising from the carrier injection at interfaces and on their transport across the layer’s bulk. In the first case, HOMO/LUMO levels are essential; in the second case, we need to deal with the electrical mobilities and the layer’s thickness.

Both hosts have a higher first excited T_1_ than DMOC-DPS, which is an advantage for a possible charge transfer to the TADFs first excited triplet level. Moreover, from the energy diagram (Figure 1a), if TmPyPb can act as an efficient hole blocking layer independently of the host, only DPEPO can be an efficient electron blocking layer resulting in a more well-designed device structure. In the first approximation, this evidence can explain the low turn-on voltage of devices with mCP as host but the better brightness when DPEPO is used. More complex is the roll-off at very high L and J ranges. As usually pointed, accumulation of T_1_ exciton in active layer leads to a strong TTA and a possible singlet-triplet annihilation (STA) with the consequent strong roll-off. Unfortunately, a long time window is required for an efficient RISC process in TADF emitters. Achieving high EQE is not a problem with a very low J (and the widely-reported results confirm such behavior); as J increases, the only way to suppress the roll-off is guaranteeing a suitable control between the T_1_ exciton and RISC process probability. Playing with molecular structure is possible, although no suitable deep-blue TADF emitter has been obtained that can be employed in a simple device structure with high EQE/low roll-off. In our work, we focus on J control. Achieving desired value can be made (besides HOMO/LUMO levels already discussed) with correct modulation of electrical charge mobility.

One of the more complicated questions in a host:guest material is the determination of the electrical carrier mobility. The most accepted model was initially proposed by Liu et al. based on the concept of a bulk-heterojunction layer [40]. They verified that the electron/hole mobilities in the host-guest system show a power dependence on the concentration of the individual materials and consider, based on the experimental results, that an electrical carrier in the matrix can be given by $\mu_{mix}=\mu_{1}^{C_{1}}\times \mu_{2}^{C_{2}}$ where index 1, 2 corresponds to the different materials and *C* is the concentration. The expression is valid for both electrons and holes, considering that *C*_1_ + *C*_2_ is equal to one. Moreover, it is also known that the presence of a D-A material (TADF) in a host material tends to alter the overall mobility for both electrons and holes [41,42]. This behavior is effectively one of the most important factors to consider in a host material for an efficient device regardless of the energy levels. In EML based on n-type DPEPO hosts, the difference of hole and electron mobilities is lower compared to the EML based on p-type mCP hosts, which induces the ability to exciton conformation, highest exciton density and better recombination in EML. This result in a much more balanced device [43]. A more adjustable electrical carrier injection/transport was achieved, leading to a better equilibrium between long life T_1_ excitons and RISC time. In the fabricated devices, not only was one of the best results for deep-blue DMOC-DPS TADF obtained, but also, as far as our knowledge goes, the best performance in roll-off point of view was effectively achieved. Moreover, EQE of 2% for brightness over 8000 cd/m^2^ were effectively the best results. The primary importance of these results, is that this simplest deep-blue OLED structure based on DMOC-DPS TADF appears to be an exciting model for further development.

## 4. Conclusions

The effect of the host environment on 10 wt.% deep-blue DMOC-DPS TADF based OLEDs has been successfully demonstrated in a simple device structure. It has been found that the performance is dependable on how the electrical properties of the host material influence the balance between charge accumulation in excited states and the RISC process. The device with the less polar host (mCP) showed a maximum ƞ_C_ of 4.12 cd/A compared to the more polar host DPEPO where ƞ_C_ was 5.77 cd/A. Both the simple structure devices exhibited a good EQE of 4% and 2.5% for DPEPO and mCP at 1000 cd/m^2^, respectively, but more interestingly, both devices showed a noticeable roll-off suppression at highest brightness, which, in the case of DPEPO host, was higher at 8185 cd/m^2^. Both kinds of devices showed lower turn voltage and excellent color stability even at high brightness. The OLED structure was the simplest found for a deep-blue TADF emitter with these characteristics. Our study helps to understand the properties of host material for excellent device fabrication. This simple device structure for deep-blue emitters can be easily implemented for fabrication in a large area continuous deposition system, which is particularly interesting for practical applications.

## Figures and Tables

**Table 1:** Summary of results obtained for structure ITO/PEDOT:PSS (40 nm)/TCTA (10 nm)/EML (20 nm)/TmPyPb (40 nm)/Cs_2_CO_3_ (2 nm)/Al (100 nm) in host bis[2-(diphenylphosphino) phenyl] ether oxide (DPEPO) and 1,3-bis(*N*-carbazolyl)benzene (mCP) hosts.

Host	ƞ_c_ (cd/A)	ƞ_p_ (lm/W)	Max EQE (%)	EQE (%) at 1000 cd/m^2^	L_max_ (cd/m^2^)/EQE (%)	Roll-Off (EQE at 1000 cd/m^2^ over Max EQE)	Roll-Off (EQE at Max L over Max EQE)
DPEPO	5.77	2.6	4.0	4.0	8185/2.0	1.0	0.5
mCP	4.12	1.7	2.63	2.5	5338/1.53	0.95	0.58
